# Mode of Delivery in Greece: A Study of Obstetricians’ Personal Preferences Regarding Delivery of Their Offspring

**DOI:** 10.3390/jcm14072444

**Published:** 2025-04-03

**Authors:** Panagiotis Christopoulos, Ermioni Tsarna, Anna Eleftheriades, Ilias Korompokis, Grigorios Karampas, Nikos F. Vlachos

**Affiliations:** Second Department of Obstetrics and Gynaecology, Aretaieion University Hospital, National and Kapodistrian University of Athens, 76 Vasilisis Sofias Avenue, 11528 Athens, Greece; ermioni.tsarna@gmail.com (E.T.); annielefth-28@hotmail.com (A.E.); mdkorompokis@gmail.com (I.K.); karampasgr@yahoo.gr (G.K.); nikosvlahos@med.uoa.gr (N.F.V.)

**Keywords:** mode of delivery, caesarian section, physicians’ preference, normal labor

## Abstract

**Background**: In Greece, the cesarean section (CS) rate reached 62.15% in 2023. This study aims to document Greek obstetricians’ preferences and choices regarding the delivery mode of their own children. **Methods**: A questionnaire was emailed to Greek obstetricians, capturing demographics, preferred and actual delivery modes, regrets about delivery choices, and opinions on factors contributing to the high CS rate. **Results:** Of the 337 respondents, 78.8% preferred normal labor, but only 55.8% reported a vaginal delivery for their first child. Only 31% would opt for vaginal birth after CS. Male and married obstetricians were more likely to prefer vaginal birth, while those with more children or children born earlier were more likely to have delivered vaginally their first child. Partner preference influenced both the obstetrician’s choice and the actual delivery mode. According to Greek obstetricians, the primary reasons for the high CS rate are hostile medico-legal conditions (56.3%), advanced maternal age and in vitro fertilization (42.6%), and lack of training in instrumental deliveries (37.2%). Maternal request was cited by 25% of respondents. **Conclusions:** Although four in five Greek obstetricians favor normal labor for their own children, the CS rate among them mirrors that of the general population. Convenience scheduling does not appear to drive Greece’s high CS rate. Obstetricians suggest that legislative reforms, improved training, and public health strategies to reduce maternal CS requests are essential for lowering the CS rate.

## 1. Introduction

Caesarian section (CS) is a lifesaving intervention, reducing maternal and neonatal mortality at the population level, when rates remain below 10% [1]. The World Health Organization recommends a CS rate of 10–15% for country-level statistics [2], though rates of 19–20% have also been proposed [3,4]. CS rate is estimated to be 21.1% worldwide, 25.7% in Europe, and 30.1% in southern Europe [1]. In Greece, after a steady increase during the last 5 years, the CS rate reached 62.15% in 2023 [5].

Although the optimal CS rate remains debated, there is consensus that some regions face limited access while others experience excessive use [1]. Maternal request contributes to the increase in CS rates due to fear of pain, pelvic floor injury, urinary incontinence, long-term sexual dysfunction, and the perception of CS as safer [6]. Additional reasons include convenience, choosing date of birth, prior negative labor experiences, and concomitant tubal ligation [6]. However, only 15% of women prefer CS, dropping to 10% among those without previous caesarian [7]. Physician-led influence also play a role, driven by fear of legal consequences, negative publicity, career risks from complications during vaginal delivery, and convenience [6]. Healthcare system factors further contribute, with caesarians more common in private than public hospitals [6,8]. Lastly, lack of training in instrumental vaginal delivery and maternal mistrust in labor care are linked to higher caesarian rates [6].

This study aims to record obstetricians’ attitudes, preferences, and choices regarding the delivery mode of their own children in Greece. These findings clarify factors contributing to high caesarian rates and obstetrician-led influences on delivery decisions.

## 2. Methods

This cross-sectional descriptive study targeted obstetricians in Greece. From July 2019 to January 2022, the Hellenic Society of Obstetrics and Gynecology (EMGE) and local Medical Associations emailed specially designed questionnaires to all professionally active and registered obstetricians. Due to multiple distribution channels, the exact number of invitations could not be determined. Submission of a single response per obstetrician was ensured by using unique birth dates and email addresses. To evaluate the representativeness of our sample and assess the potential impact of any deviations on our findings, we compared the sex distribution and professional status of our study participants with published statistical data on all 2428 obstetricians in Greece [9]. To this end, Fisher’s Exact Test was applied.

The study questionnaire covered demographics, delivery preferences, actual delivery mode, regrets (for participants with children), and opinions regarding the underlying reasons for Greece’s high CS rates. Female obstetricians answered about their pregnancies, while males referred to their partners’. Demographics included birth date, sex, work area, professional status (private practice/university hospital/national healthcare system/other), marital status (married/single/divorced/other), number of pregnancies and children, ongoing pregnancy (yes/no), and gestational age for ongoing pregnancies. Delivery preferences included the obstetrician’s and their partner’s preferred delivery mode (normal labor/caesarian). For participants with children, data collected included child’s birth year (1970–1980, 1981–1990, 1991–1995, 1996–2000, 2001–2005, 2006–2010, 2011–2015, after 2015), gestational age at birth, birth weight, delivery mode, caesarian indication (free text) and whether it was elective (yes/no), and use of epidural analgesia for normal labor. These questions were repeated for each child if applicable. Regret-related questions asked participants about delivery preference if given the choice again, regrets for delivery mode (yes/no), willingness to have a vaginal birth after caesarian delivery (VBAC), and their most negative delivery-related experiences (free text). Lastly, obstetricians chose up to two causes for the high caesarian rates in Greece among the following: maternal request, lack of education on instrumental delivery, advanced maternal age/use of in vitro fertilization (IVF), unclear/hostile medico-legal framework (defensive medicine), complicated pregnancies/lack of antenatal tests, non-compliance with guidelines, conflict between guidelines and clinical experience, increased detection of complications/congenital anomalies due to technological advances, subjective fetal heart monitoring interpretation, and other reasons.

For descriptive statistics, we used median and interquartile range (IQR) for continuous variables (due to non-normality) and frequencies with proportions for categorical variables. We compared participant characteristics by sex, professional status, and prior labor history (yes/no) using the Chi-square test for categorical variables and the Kruskal–Wallis Rank Sum Test for continuous variables. Similar comparisons were performed for participants with children based on the year of their first child’s birth. For preferred delivery mode, univariate logistic regression models were built using as independent variables obstetrician’s birth year, sex, professional status, marital status, number of pregnancies, number of children, ongoing pregnancy, and partner’s delivery preference. Significant variables from univariate models were included in a multivariable logistic regression model, incorporating the number of pregnancies rather than the number of children (due to collinearity). For participants with children, a similar analysis was conducted for the delivery mode of their first child, adding the child’s birth year as an independent variable. Lastly, the Chi-square test and Kruskal–Wallis Rank Sum Test compared participants’ characteristics by first child’s delivery mode regret, separately for normal labor and caesarian delivery.

Statistical analyses were conducted using R (Version 1.4.1106) with the packages “clipr”, “Hmisc”, and “tableone” [10,11,12,13]. A 5% significance level was applied to all tests. The study was conducted under the auspices of EMGE.

## 3. Results

A total of 337 obstetricians participated in the study. Of these, 105 identified as female and 230 as male (Table 1). The median birth year was 1972 (IQR 1964–1978). Participants reported a median of two pregnancies and two births, though 44 (13.1%) had no children. Regarding professional status, 69% worked in the private sector, 14.6% in the national healthcare system, 7.4% in university hospitals, and 8.9% in other roles (e.g., Hellenic National Public Health Organization, non-governmental organizations). Our study population represents 13.9% of all obstetricians in Greece. Women constitute 31.3% of our sample compared to 16.7% among all Greek obstetricians, leading to a significant difference (*p* < 0.01). Additionally, a significant disparity was observed in professional status (*p* < 0.01), as university-affiliated obstetricians were over-represented in our study population (7.4% vs. 4.2%).

Among the 337 study participants, 9.9% were single, 85.3% were married, and 4.8% were divorced. Only 2.7% reported an ongoing pregnancy. Although 78.8% preferred normal labor, only 55.8% of those with children reported their first child was delivered vaginally. Additionally, 72.8% of obstetricians’ partners preferred normal labor. The median gestational age at first delivery was 39 weeks (IQR 38–40), and the median birth weight was 3200 g (IQR 2965–3500). Most births (62.9%) occurred after 2000. Negative experiences with normal labor included vaginal/perineal tears (n = 19), pain (n = 19), hemorrhage (n = 16), and neonatal morbidity (n = 15). For cesarean deliveries, negative experiences included pain (n = 21), surgical complications (n = 20), hemorrhage (n = 19), and neonatal morbidity (n = 1). Taking into account these experiences, 60.2% said they would still choose normal delivery. However, only 31% (n = 104) would opt for VBAC for themselves or their female partners.

Several significant differences emerged between female and male obstetricians (Table 1). Female obstetricians were younger (*p* < 0.01), more often single or divorced (*p* < 0.01), had fewer pregnancies (*p* < 0.01) and children (*p* < 0.01), and their children’s births occurred more recently (*p* < 0.01). Professionally, females were less likely to work in university hospitals or the national healthcare system and more likely to work in the private sector or other roles (*p* < 0.01). Normal labor was preferred by 63.4% of female obstetricians compared to 86.6% of males (*p* < 0.01). Partner preference (*p* = 0.91) and first child’s delivery mode (*p* = 0.44) showed no sex differences, but female obstetricians had lower gestational age at birth (*p* = 0.02). Negative experiences differed by sex for both normal (*p* = 0.04) and cesarean delivery (*p* = 0.03). Females reported pain more often after normal labor and less often hemorrhage, vaginal/perineal tears, and neonatal morbidity. After cesarean delivery, females more often cited pain and surgical complications, but cited hemorrhage less often.

Regarding professional status, older participants were more likely to work in university hospitals, followed by private practice, the national healthcare system, and other roles (*p* < 0.01). Men were more likely to work in university hospitals, followed by the national healthcare system, private practice, and other roles (*p* < 0.01). University hospital employment correlated with the highest number of pregnancies and children, while the lowest was seen in those with other professional roles (*p* < 0.01 for both). However, professional status was not found to be associated with either the obstetrician’s preferred mode of delivery (*p* = 0.86) or the mode of delivery of the obstetrician’s first child (*p* = 0.47). Participants without children were younger (*p* < 0.01), more likely to be female (*p* = 0.03), and more often single (*p* < 0.01). They tended to work in the national healthcare system or other roles rather than university hospitals or private practice (*p* < 0.01) and were less likely to prefer normal labor (*p* = 0.04). Participants whose first child was born more recently were younger (*p* < 0.01) and reported fewer pregnancies (*p* = 0.01) and children (*p* < 0.01). Obstetrician sex also differed by the first child’s birth year (*p* < 0.01). Preference for normal labor declined in more recent childbirths from 100% (1970 to 1980) to 62.5% (post-2015) (*p* = 0.046). Similarly, normal labor rates dropped from 93.8% (1970–1980) to only 28% (post-2015) (*p* < 0.01).

In the univariate analysis of obstetricians’ delivery preferences, professional status (*p* = 0.85), and ongoing pregnancy (*p* = 0.55) were not significant (Table 2). Higher odds for preferring caesarian delivery were observed in younger obstetricians (OR = 1.04) and those whose partners preferred caesarian delivery (OR = 26.41). Conversely, lower odds were observed for male (OR = 0.27) and married participants (OR = 0.20), and those with more pregnancies (OR = 0.79) and children (OR = 0.65). In the multivariable analysis, the year of the obstetrician’s birth and number of pregnancies were no longer significant (Table 2). Male obstetricians were less likely to prefer caesarian delivery (OR = 0.13; 95% CI: 0.04–0.38), while the partner’s preference for caesarian delivery significantly increased the odds (OR = 66.23; 95% CI: 21.96–260.64). Marital status also influenced preference, with married participants having lower odds than single participants (OR = 0.10; 95% CI: 0.02–0.39).

In the univariate analysis of the first child’s delivery mode among 293 participants with children, sex (*p* = 0.32), professional (*p* = 0.36) and marital status (*p* = 0.06), and ongoing pregnancy (*p* = 0.28) were not significant (Table 3). Higher odds for caesarian delivery were found in younger obstetricians (OR = 1.06), those with partners preferring caesarian delivery (OR = 17.20), and for children born later (*p* < 0.01). For births after 2005, ORs were greater than 1, while for those before 2001, the value was lower than 1, using 2001–2005 as a reference. Lower odds were observed in obstetricians with more pregnancies (OR = 0.62) and children (OR = 0.38). In the multivariable analysis, the obstetrician’s birth year was no longer significant (*p* = 0.15). Caesarian delivery odds decreased with more pregnancies (OR = 0.58; 95% CI: 0.39–0.83) but increased with partner’s preference for caesarian (OR = 27.07; 95% CI: 10.05–89.04) and children born more recently (*p* = 0.01) (Table 3).

Of the 163 obstetricians whose first child was vaginally delivered, 149 responded regarding delivery regrets, with only six (4%) expressing regret—three men (50%) and three women (50%) (Table S1). Compared to those without regrets, this group reported more negative experiences, such as neonatal morbidity, pain, vaginal/perineal tears, and hemorrhage (*p* < 0.01). Their children had a higher median birth weight (3600 g vs. 3200 g, *p* = 0.02), and epidural analgesia was not used (*p* = 0.04). Additionally, 83.3% would opt for a CS if given the choice again (*p* < 0.01). No significant differences were found for year of birth (*p* = 0.21), sex (*p* = 0.46), professional (*p* = 0.53) or marital status (*p* = 0.84), number of pregnancies (*p* = 0.08), children (*p* = 0.75), or gestational age (*p* = 0.66).

Of the 129 obstetricians whose first child was delivered via CS, 121 responded about delivery regrets. Only seven (5.8%) expressed regret—four men (57.1%) and three women (42.9%) (Table S2). Compared to those without regrets, no significant differences were found in sex (*p* = 0.81), professional (*p* = 0.05) or marital status (*p* = 0.68), number of children (*p* = 0.87), child’s birth year (*p* = 0.65), birth weight (*p* = 0.07), gestational age (*p* = 0.91), CS indication (*p* = 0.29), or negative CS experiences (*p* = 0.85). However, those who regretted CS were younger (*p* = 0.04), had more pregnancies (*p* = 0.02), and all would opt for normal labor if given the choice again (*p* < 0.01). However, willingness to attempt VBAC was similar between groups (*p* = 0.08).

Greek obstetricians identified several key factors contributing to the high CS rate (Table 4). The leading cause, cited by 189 (56.3%) participants, was the unclear or hostile medico-legal framework, promoting defensive medicine. Additionally, 104 (31%) noted non-compliance with medical guidelines, 62 (18.5%) pointed to the subjective interpretation of fetal heart monitoring, and 26 (7.7%) mentioned conflicts between guidelines and clinical experience. Regarding healthcare system factors, 125 (37.5%) highlighted insufficient training in instrumental delivery, and 84 (25%) cited maternal request for CS. A genuine rise in medically indicated CS was also suggested, with 143 (42.6%) attributing it to advanced maternal age and IVF, 75 (22.3%) to better detection of pregnancy complications and anomalies due to technological advances, and 19 (5.7%) to more complicated pregnancies or lack of antenatal testing.

## 4. Discussion

In this study of 337 Greek obstetricians, 78.8% preferred vaginal delivery for their own children, but this dropped to 55.8% vaginal delivery of their first child. Only 31% would opt for VBAC. Male and married obstetricians were more likely to prefer vaginal birth, while having more children and earlier births correlated with actual vaginal delivery. There was a tendency for the partner’s preference to align with the obstetrician’s choice and the delivery mode. Over half (56.3%) cited the hostile medico-legal environment as the primary reason for high cesarean rates in Greece. Advanced maternal age and IVF were noted by 42.6% and lack of training in instrumental delivery by 37.2%. A non-negligible 25% identified maternal request as a contributing factor.

This study offers key insights into decision-making processes of obstetricians in Greece, being the first to explore how medical experiences, risk assessments, and cultural or systemic factors influence their delivery preferences. By examining their personal and professional views on childbirth, the study highlights factors such as safety perceptions, legal concerns, convenience, and pressures from patients or healthcare systems. The findings reveal potential barriers to promoting vaginal births, including gaps in medical training, inadequate patient education, and systemic issues. Addressing these barriers could lead to more balanced, patient-centered approaches to childbirth that respect both medical expertise and patient choice. We acknowledge that our study population differed from the broader population of obstetricians in Greece with respect to sex and professional status. However, the mode of delivery of the obstetrician’s first child in our study was not associated with either sex or professional status, suggesting that our results are generalizable to the wider population of Greek obstetricians. Regarding obstetricians’ preferred mode of delivery, we found that women, who are over-represented in our study, were less likely to prefer normal labor. Therefore, the actual preference for normal labor among Greek obstetricians is likely higher than the 78.8% recorded in our study, further suggesting that Greece’s high CS rate is unlikely to be primarily driven by obstetricians’ personal preference for mode of delivery. This study is significant given that Greece’s CS rate exceeded 62% in 2023, one of the highest globally. Elevated rates increase risks like maternal morbidity, prolonged recovery, and complications in future pregnancies, making this a public health priority. While vaginal birth is generally preferred, CS is beneficial in high-risk cases, such as placental disorders, preeclampsia, diabetes, multiple pregnancies, abnormal fetal presentation, or previous CS. Cesareans can also prevent pelvic floor injuries, urinary incontinence, and pelvic organ prolapse, and reduce the need for emergency interventions like instrumental delivery, which may pose risks to both mother and baby [14].

Considering the influence of physician preference, several studies worldwide have explored obstetricians’ attitudes toward the delivery mode of their own children. Notably, results from studies conducted in different geographical regions are expected to reflect cultural differences in delivery preferences and differences in healthcare practices. Our study in Greece shows a 21.2% preference for CS, whereas higher rates have been reported in Turkey, China, and Iran, and lower rates in Denmark, Germany, the UK, and Ireland [15,16,17]. Notably, CS rates exceed 30% in Western and Eastern Asia (Turkey and China) and are significantly lower in Northern and Western Europe (Denmark, UK, Ireland, and Germany) [18]. This suggests a link between obstetricians’ preferences and population-level cesarean rates, independent of physician-led influences due to the medico-legal framework or convenience, as these do not apply to their own childbirth decisions. Indeed, obstetricians cite concerns about pelvic floor injury, sexual dysfunction, incontinence, pelvic organ prolapse, and fetal risks as reasons for preferring CS over vaginal delivery [15,16,17,19]. However, it remains unclear how these personal fears influence the provision of evidence-based information to support women’s informed choices regarding childbirth [14].

Our study identified defensive medicine driven by an unclear or hostile medico-legal framework as the primary factor behind Greece’s high CS rates. Although no data exist on delivery methods in Greek legal disputes, studies from other countries show a higher risk of lawsuits related to vaginal delivery, with obstetricians perceiving CS as safer in the context of potential legal consequences [6,20,21,22]. In the USA, obstetricians with a history of lawsuits tend to perform more often CS, particularly for patients of lower socioeconomic status [6,20]. Additionally, frequent worry about lawsuits is associated with recommending more cesarean deliveries [23]. In Romania, 69.9% admitted to performing defensive CS, and 86.3% reported that fear of malpractice influenced their counseling on delivery mode. Similarly, Italian obstetricians altered their practices due to legal fears, particularly regarding accusations of failing to perform or delaying CS, rather than concerns over surgical complications [22]. A Portuguese study found perinatal asphyxia in 50% of lawsuits, neonatal trauma in 24%, maternal complications in 19%, and potential prenatal diagnostic errors in 5.54% of lawsuits [20]. Neonatal trauma was often linked to instrumental deliveries, shoulder dystocia, or breech presentation [20]. In 68.7% of perinatal asphyxia cases, obstetricians were accused of not performing or delaying a CS [20]. Thus, fear of legal consequences may underlie other reasons for increased cesarean rates in Greece. For example, reluctance to perform instrumental deliveries may reflect both defensive medicine and inadequate training. Similarly, opting for immediate CS in cases of non-reassuring fetal heart patterns may be influenced by defensive medicine and subjective interpretation [24,25].

In our study, 21.2% of obstetricians declared a preference for caesarian delivery of their children, while 44.2% of obstetricians’ first children were actually born via caesarian section. Considering the birth year of obstetricians’ first child, the reported cesarean delivery rate among obstetricians’ children is comparable to, if not higher than, that of the general population, despite the reported 78.8% preference for normal labor. This discrepancy between preferred and actual delivery mode highlights the significant influence of safety perceptions, medical indications, and educational gaps on cesarean section rates, independent of the medico-legal framework, which is unlikely to impact the delivery mode for obstetricians’ children.

In our study, 42.6% of obstetricians cited advanced maternal age and IVF, while 22.3% cited pregnancy complications as reasons for Greece’s high CS rate. This suggests that a genuine increase in medically indicated cesareans is regarded as plausible by the obstetricians. A maternal age over 35 is associated with chromosomal abnormalities, congenital malformations, and adverse outcomes [25]. For mothers over 40, risks for small- and large-for-gestational-age infants also rise, along with comorbidities like diabetes, hypertension, and obesity [26]. Although advanced maternal age alone is not an absolute indication for cesarean delivery, it is considered a risk factor, and delivery at 39–39+6 weeks is recommended for women over 40 [26]. Notably, the impact of labor induction on CS rates remains debated [27,28,29,30,31]. In Greece, the average maternal age at first birth increased from 27.9 years in 2002 to 31 years in 2022, and births to mothers over 40 rose from 2.5% to 10% in the same period [32]. Given Greece’s low fertility rate (1.32 live births per woman in 2022), the increased risk of placenta accreta and hysterectomy due to multiple cesareans may affect only a minority of women [32,33]. This demographic shift among expectant mothers likely contributes to the rising cesarean rates in Greece.

Lack of education on instrumental delivery was identified by obstetricians in our study as a key factor in the rising CS rate. Although CS after labor onset are linked to more maternal and neonatal complications than vaginal births, the long-term cognitive outcomes for children born via non-planned cesarean are similar to those born by instrumental delivery, though lower than those born by non-instrumental vaginal delivery complications of instrumental vaginal delivery include maternal injuries (anal sphincter damage, cervical tears, and severe vaginal or labial tears) and neonatal morbidity (scalp or facial injuries, neonatal intensive care unit admission) [34,35]. Globally, instrumental deliveries have decreased alongside rising cesarean rates, with a sharper decline in forceps deliveries compared to vacuum deliveries, although forceps are still more likely to result in vaginal delivery [25,34,36,37]. While the need for obstetricians’ training in instrumental deliveries is widely acknowledged, concerns about insufficient cases for adequate training persist [36,37]. In the UK, a postgraduate educational program using video material, dolls, and pelvis models did not improve success rates but did reduce maternal and neonatal morbidity [35]. Apart from unmet educational needs, fear of legal consequences may hinder increased use of instrumental deliveries, as these procedures are safest when performed by experienced professionals, potentially discouraging senior obstetricians from allowing trainees to perform them, even under supervision [24,34].

Lastly, obstetricians in Greece noted that maternal request significantly contributes to the high CS rate. A systematic review found that 3% of all deliveries (range 0.2–42%) and 11% of all cesareans (range 0.9–60%) are performed without medical indications, solely due to maternal request [38]. This is more common after 2010 and in upper-middle-income countries [38]. Another review estimated that 5–20% of women in high-income countries and 1.4–50% in low-middle-income countries prefer cesarean delivery [39]. It is apparent that both maternal preference for cesarean delivery and caesarian rates due to maternal request vary widely by study and country. Factors associated with caesarian preference include first trimester delivery decision, smoking, young age, nulliparity, lower educational level, depressive symptoms, negative birth experiences, and fear of pain and physical damage [7,40,41,42]. Conversely, women with more knowledge about normal labor physiology are less likely to request a CS [43]. However, in our study, 36.6% of female obstetricians who have a high educational level and substantial knowledge on labor physiology expressed a preference for caesarian delivery, a figure significantly higher than the 5–20% observed among women in high-income countries [39]. This suggests that maternal request may be even more prevalent in the Greek general population, supporting obstetricians’ view that maternal request is a significant contributor to the high cesarean section rate in Greece. A systematic review identified three main reasons for maternal request: social norms (social influence, culture, and choice), emotional experiences (fear, safety and risk perception, control, and avoidance of memory), and personal experiences (regarding prior birth and healthcare encounters) [44].

The obstetricians’ compliance with maternal requests for CS further affects the impact of maternal preferences on CS rates. A study across eight European countries found that obstetricians’ compliance with maternal requests for cesareans ranged from 15% in Spain to 79% in the UK, influenced by country, fear of legal dispute, and university hospital affiliation, while female obstetricians with children were least likely to comply [45]. To be noted, birth experiences are similar between women with vaginal delivery and those with cesarean on request, while emergency cesarean or instrumental vaginal delivery are associated with more negative experiences [46]. In conclusion, the rate of cesareans due to maternal request depends on both the proportion of women preferring cesareans and obstetricians’ compliance. Given the perceived hostile medico-legal framework in Greece, high obstetrician compliance may explain the significant role of maternal request in the country’s elevated CS rate.

## 5. Conclusions

In this survey of Greek obstetricians, one in five preferred CS for their own children, yet the rate of normal labor was similar to or lower than the general population, considering the child’s birth year. Our results suggest that scheduling births for convenience does not significantly contribute to the high cesarean rate in Greece, despite common public perceptions. Obstetricians indicated that legislative changes and addressing educational gaps are necessary to increase normal labor rates. Additionally, public health strategies should address maternal requests for cesarean, while respecting women’s autonomy. These findings support initiatives to promote evidence-based, patient-centered care, ensuring obstetricians are equipped to advocate for safe vaginal births when appropriate.

## Figures and Tables

**Table 1:** Descriptive characteristics of the total study population (n = 337), grouped by sex (n = 105 females, n = 230 males, n = 2 missing values). IQR: interquartile range.

		Total Study Population (n = 337)	Female Sex (n = 105)	Male Sex (n = 230)	*p*-Value (Male vs. Female Sex)
Year of birth, median (IQR)		1972 (1964, 1978)	1975(1968, 1981)	1969 (1961, 1976)	<0.01
Number of pregnancies, median (IQR)		2 (1, 3)	2 (1, 2)	2 (1, 3)	<0.01
Number of children, median (IQR)		2 (1, 2)	2 (1, 2)	2 (1, 2)	<0.01
Proffessional Status, n (%)	Private practice	232 (69%)	77 (73.3%)	154 (67.2%)	<0.01
	National healthcare system	49 (14.6%)	10 (9.5%)	38 (16.6%)	
	University hospital	25 (7.4%)	2 (1.9%)	23 (10.0%)	
	Other	30 (8.9%)	16 (15.2%)	14 (6.1%)	
Marital Status, n (%)	Single	33 (9.9%)	20 (19.2%)	13 (5.7%)	<0.01
	Married	284 (85.3%)	75 (72.1%)	207 (91.2%)	
	Divorced	16 (4.8%)	9 (8.7%)	7 (3.1%)	
	Other	0 (0%)	0 (0%)	0 (0%)	
Mode of delivery preference, n (%)	Normal labor	246 (78.8%)	64 (63.4%)	181 (86.6%)	<0.01
	Caesarian Delivery	66 (21.2%)	37 (36.6%)	28 (13.4%)	
Partner’s mode of delivery preference, n (%)	Normal labor	217 (72.8%)	63 (71.6%)	152 (73.1%)	0.91
	Caesarian Delivery	81 (27.2%)	25 (28.4%)	56 (26.9%)	
Mode of 1st child’s delivery, n (%)	Normal labor	163 (55.8%)	44 (51.8%)	118 (57.6%)	0.44
	Caesarian Delivery	129 (44.2%)	41 (48.2%)	87 (42.4%)	
Gestational age at 1st child’s birth in weeks, median (IQR)		39 (38, 40)	39 (38, 39.7)	39 (38.25, 40)	0.02
Birthweight of 1st child, median (IQR)		3200 (2965, 3500)	3150 (2910, 3557.5)	3200 (3000, 3452.5)	0.66
Year of 1st child’s birth, n (%)	1970–1980	16 (5.5%)	1 (1.2%)	15 (7.4%)	<0.01
	1981–1990	25 (8.6%)	3 (3.5%)	22 (10.8%)	
	1991–1995	26 (8.9%)	8 (9.4%)	18 (8.8%)	
	1996–2000	41 (14.1%)	8 (9.4%)	33 (16.2%)	
	2001–2005	40 (13.7%)	21 (24.7%)	19 (9.3%)	
	2006–2010	52 (17.9%)	19 (22.4%)	32 (15.7%)	
	2011–2015	66 (22.7%)	19 (22.4%)	47 (23.0%)	
	After 2015	25 (8.6%)	6 (7.1%)	18 (8.8%)	
Ongoing pregnancy, n (%)	No	328 (97.3%)	104 (99.0%)	222 (96.5%)	0.34
	Yes	9 (2.7%)	1 (1.0%)	8 (3.5%)	

**Table 2:** Results from simple and multiple logistic regression models with the dependent variable representing the obstetrician’s preferred mode of delivery (n = 337). REF: reference, NA: non applicable.

		Univariate Logistic Regression Models, OR (95%CI)	*p*-Value	Multivariable Logistic Regression Model, OR (95%CI)	*p*-Value
Year of birth		1.04 (1.01, 1.08)	0.01	0.99 (0.93, 1.06)	0.79
Sex	Female	REF	<0.01	REF	<0.01
	Male	0.27 (0.15, 0.47)		0.13 (0.04, 0.38)	
Proffessional Status	Private practice	REF	0.85	NA	NA
	National healthcare system	0.70 (0.27, 1.59)			
	University hospital	1.06 (0.33, 2.84)			
	Other	1.09 (0.41, 2.59)			
Marital Status	Single	REF	<0.01	REF	<0.01
	Married	0.20 (0.09, 0.43)		0.10 (0.02, 0.39)	
	Divorced	1.06 (0.32, 3.55)		0.30 (0.03, 4.14)	
Number of pregnancies		0.79 (0.63, 0.99)	0.04	1.23 (0.84, 1.83)	0.29
Number of children		0.65 (0.49, 0.84)	<0.01	NA	NA
Ongoing pregnancy		0.53 (0.03, 3.03)	0.55	NA	NA
Partner’s mode of delivery preference	Normal labor	REF	<0.01	REF	<0.01
	Caesarian Delivery	26.41 (12.87, 58.54)		66.23 (21.96, 260.64)	

**Table 3:** Results from simple and multiple logistic regression models with the dependent variable representing the mode of delivery of the obstetrician’s first child (*n* = 293). REF: reference, NA: non applicable.

		Univariate Logistic Regression Models, OR (95%CI)	*p*-Value	Multivariable Logistic Regression Model, OR (95%CI)	*p*-Value
Year of birth		1.06 (1.03, 1.09)	<0.01	0.94 (0.87, 1.02)	0.15
Sex	Female	REF	0.32	NA	NA
	Male	0.77 (0.46, 1.29)			
Proffessional Status	Private practice	REF	0.36	NA	NA
	National healthcare system	0.80 (0.39, 1.61)			
	University hospital	0.69 (0.28, 1.61)			
	Other	2.05 (0.74, 6.22)			
Marital Status	Single	NA	0.06	NA	NA
	Married	REF			
	Divorced	1.53 (0.53, 4.48)			
Number of pregnancies		0.62 (0.48, 0.78)	<0.01	0.58 (0.39, 0.83)	<0.01
Number of children		0.38 (0.26, 0.53)	<0.01	NA	NA
Ongoing pregnancy		2.56 (0.49, 18.68)	0.28	NA	NA
Partner’s mode of delivery preference	Normal labor	REF	<0.01	REF	<0.01
	Caesarian Delivery	17.20 (8.35, 39.34)		27.07 (10.05, 89.04)	
Year of 1st child’s birth	1970–1980	0.07 (0.00, 0.41)	<0.01	NA	0.01
	1981–1990	0.04 (0.00, 0.23)		0.02 (0.00, 0.26)	
	1991–1995	0.30 (0.09, 0.87)		0.40 (0.07, 1.99)	
	1996–2000	0.58 (0.23, 1.39)		0.19 (0.04, 0.76)	
	2001–2005	REF		REF	
	2006–2010	1.26 (0.55, 2.90)		0.96 (0.27, 3.52)	
	2011–2015	1.44 (0.66, 3.20)		2.36 (0.64, 9.21)	
	After 2015	2.57 (0.91, 7.88)		3.43 (0.61, 20.69)	

**Table 4:** Contributing reasons to the high caesarian section rates in Greece, according to the 336 study participants, in declining order.

Reasons for High Caesarian Section Rates in Greece	n (%)
Unclear/hostile medico-legal framework (defensive medicine)	189 (56.3%)
Advanced maternal age at birth of first child and use of in vitro fertilization (IVF)	143 (42.6%)
Obstetricians’ lack of education regarding instrumental delivery	125 (37.2%)
Non-compliance with medical guidelines	104 (31%)
Maternal request	84 (25%)
Increase in detection of pregnancy complications and congenital anomalies due to technological advances	75 (22.3%)
Subjective interpretation of fetal heart monitoring	62 (18.5%)
Other reasons	40 (11.9%)
Conflict between medical guidelines and obstetrician’s clinical experience	26 (7.7%)
Increase in complicated pregnancies or pregnancies without antenatal tests performed	19 (5.7%)

## Data Availability

The raw data supporting the conclusions of this article will be made available by the authors on request.

## Supplementary Materials

The following supporting information can be downloaded at: https://www.mdpi.com/article/10.3390/jcm14072444/s1, Table S1: Descriptive statistics of participants, whose first child was born with normal labor, grouped based on regrets regarding more of delivery; Table S2: Descriptive statistics of participants, whose first child was born with caesarian section, grouped based on regrets regarding more of delivery.
